# Vascular Endothelial Growth Factor, Basic Fibroblast Growth Factor, and Pigment Epithelium-Derived Factor Expression in the Neovascular Iris in Retinal Diseases

**DOI:** 10.1155/2018/8025951

**Published:** 2018-04-11

**Authors:** Heng Miao, Xianru Hou, De-Kuang Hwang, Yong Tao

**Affiliations:** ^1^Department of Ophthalmology, People's Hospital, Peking University, Beijing, China; ^2^Department of Ophthalmology, Taipei Veterans General Hospital, Taipei, Taiwan; ^3^Department of Ophthalmology, School of Medicine, National Yang-Ming University, Taipei, Taiwan; ^4^Department of Ophthalmology, Beijing Chaoyang Hospital, Capital Medical University, Beijing, China

## Abstract

**Objective:**

To determine the expression of cytokines in the iris of patients with neovascular glaucoma (NVG).

**Methods:**

Patients with NVG associated with proliferative diabetic retinopathy (PDR, group 1) or central retinal vein occlusion (CRVO, group 2) who had undergone surgical treatment were enrolled. Patients with primary open-angle glaucoma requiring surgical treatment were included in the control group (group 3). All iris specimens were obtained during trabeculectomy, 7 days after intravitreal injections of ranibizumab. The messenger RNA (mRNA) and protein levels of three target cytokines—vascular endothelial growth factor (VEGF), basic fibroblast growth factor (bFGF), and pigment epithelium-derived factor (PEDF)—in the iris were analyzed and compared.

**Results:**

We included 39 eyes from 39 patients (12, 15, and 12 in groups 1, 2, and 3, resp.). The protein and mRNA levels of PEDF were higher in two NVG groups. The protein levels, but not mRNA level, of bFGF were higher in the two NVG groups. The protein and mRNA levels of VEGF were similar in the three groups.

**Conclusions:**

The protein level of bFGF increased in the irises of the NVG patients was not expressed by the iris itself, whereas PEDF may be expressed by the iris tissue in these patients.

## 1. Introduction

Neovascular glaucoma (NVG) is a type of glaucoma in which progressive optic nerve damage and intraocular pressure elevation are mainly caused by the mechanical blockage of the aqueous outflow of neovascularization from the iris and anterior chamber angle [1]. Compared with primary glaucoma, NVG is relatively difficult to treat, and its visual outcomes are poorer [2]. Various pro- and antiangiogenic factors and inflammatory cytokines participate in the formation of iris neovascularization, including vascular endothelial growth factor (VEGF), basic fibroblast growth factor (bFGF), and pigment epithelium-derived factor (PEDF) [3, 4].

Patients with intraocular ischemic or inflammatory conditions, such as diabetic retinopathy, retinal vein occlusion, and retinopathy of prematurity, commonly develop NVG [1, 5, 6]. In these patients, the pathogenesis of iris neovascularization may be ascribed to the anterior diffusion of cytokines that are expressed by hypoxic retinal tissues; hence, standard treatment options focus on the blockage of cytokines and the treatment of the ischemic retina, such as retinal photocoagulation and cryotherapy [3]. However, despite repeated treatments, iris neovascularization may continuously progress in clinical settings. After chronic intraocular inflammation and ischemia, the iris tissue may itself secrete various factors and cytokines to compensate for the imbalance.

In the present study, we measured the messenger RNA (mRNA) and protein levels of VEGF, bFGF, and PEDF in the iris tissue of patients who had NVG associated with retinal diseases and had undergone surgical treatment. By determining the difference between the mRNA and protein levels, we examined whether these cytokines are expressed by the iris itself in these patients to gain an in-depth understanding of the pathogenesis of NVG.

## 2. Methods

### 2.1. Study Patients

This prospective case-control study was approved by the Institutional Review Board of Peking University People's Hospital and was conducted in adherence to the tenets of the Declaration of Helsinki. Patients with NVG secondary to proliferative diabetic retinopathy (PDR, group 1) and central retinal vein occlusion (CRVO, group 2) were enrolled in this study after receiving the explanation of and signing the informed consent form. In addition, patients with primary open-angle glaucoma (POAG, group 3) were selected and enrolled in the control group. Patients' age, sex, best-corrected visual acuity, and preoperative intraocular pressure were recorded. Patients with multiple mechanisms or etiologies for glaucoma were excluded from the study.

### 2.2. Sample Collection and Measurement

Approximately 7 days before surgery, 0.5 mg of ranibizumab was intravitreally injected in all operated NVG eyes [7]. All patients underwent standard trabeculectomy with mitomycin-C. During the operation, peripheral iridectomy was performed, and the iris tissue specimen was collected. The specimen was then divided into two parts and stored at −70°C for measurement [7].

The first part of the specimen of each patient was used for determining the mRNA level in triplicate. Total RNA was extracted using the spin or vacuum total RNA isolation system (Promega Corp., Madison, USA) and reverse transcribed using a complementary DNA (cDNA) synthesis kit (RevertAid First Strand cDNA Synthesis Kit, Fermentas, Burlington, ON, Canada) [8]. Real-time polymerase chain reaction (PCR) was conducted using the Thermo Scientific PikoReal real-time PCR system (Thermo Fisher Scientific Corp., USA) with SYBR green I (GoTaq qPCR Master Mix, Promega Corp.). The average threshold cycle (Ct) values of targeted molecules were calculated on the basis of three repeated measurements for each sample [8]. Because we used the solid iris tissue, instead of soluble liquids such as the aqueous humor, as the sample in this study and because the sizes of the excised tissues varied, we used the relative amount, instead of the absolute amount, after normalization. The mRNA levels of VEGF, bFGF, and PEDF were determined and compared after being normalized to the mRNA level of glyceraldehyde-3-phosphate dehydrogenase (GAPDH) by using the 2^−△△Ct^ method. Primer sequences used in the analysis are listed in Table 1 [7].

For protein analysis, all of the remaining iris tissue specimens in each group were mixed together and compared because the amount of the remaining tissue specimens was too low to be used for determining the protein level of each growth factor individually. Each sample was mixed with 500 *μ*L of cell lysis buffer (P0013, Beyotime Biotech, Jiangsu, China) and homogenized using a glass homogenizer. After centrifugation, the supernatant was used for Western blot analysis. Before electrophoresis, the bicinchoninic acid protein assay (Beyotime Biotech, Jiangsu, China) was used and 20 *μ*g of the protein was loaded onto each lane [9]. The concentration of each cytokine protein was measured using the Western blot technique. The primary antibodies VEGF (ab1316, 1 : 200 dilution, Abcam Inc., Cambridge, MA, USA), bFGF (ab181, 1 : 250 dilution, Abcam Inc.), and PEDF (bs-0731R, 1 : 100 dilution, Bioss Co., Beijing, China) were used for the analysis. Protein levels were quantified through densitometry and normalized to the GAPDH level (Gel-Pro analyzer 4.0, Media Cybernetics, USA) [10].

### 2.3. Statistical Analyses

The baseline characteristics of the patients in the three groups were compared using the one-way analysis of variance (ANOVA) and chi-square tests. The mRNA levels in each specimen and the pooled protein level of the 3 target cytokines (VEGF, bFGF, and PEDF) were calculated 3 times. The average results of mRNA levels are reported as the mean ± standard deviation. The chi-square test and the one-way ANOVA test with Bonferroni correction were used to compare categorical and continuous variables, respectively, between the disease and control groups. All statistical analyses were performed using the Statistical Package for Social Sciences (version 19.0) for Windows (SPSS Inc., Chicago, IL, USA). The two-sided significance level was set at 0.05.

## 3. Results

At the end of enrollment, 39 eyes from 39 patients were included. Of the 39 eyes, 12, 15, and 12 were associated with PDR, CRVO, and POAG and accordingly included in the PDR, CRVO, and control groups, respectively. No significant difference in age, sex, or intraocular pressure was observed among the three groups. However, visual acuity was better in patients with POAG (Table 2).

**Figure 1:**
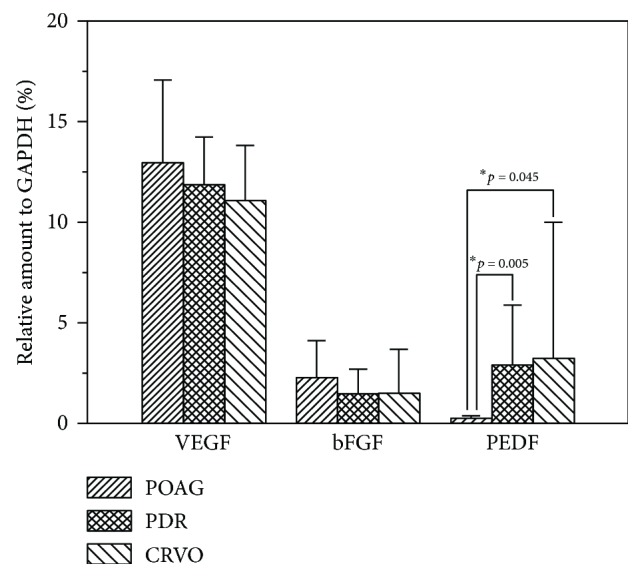
mRNA level normalized to that of glyceraldehyde-3-phosphate dehydrogenase (GAPDH) in the iris. The mRNA levels of vascular endothelial growth factor (VEGF) and basic fibroblast growth factor (bFGF) did not differ among patients with neovascular glaucoma (NVG) secondary to proliferative diabetic retinopathy (PDR), central retinal vein occlusion (CRVO), and primary open-angle glaucoma (POAG) (*p* = 0.436 and 0.621, resp.). The mRNA level of pigment epithelium-derived factor (PEDF) was significantly higher in patients with NVG secondary to PDR (*p* = 0.005) and CRVO (*p* = 0.045) than in patients with POAG. No significant difference was observed between the two NVG groups (*p* = 0.584).

The mean mRNA levels of VEGF and bFGF did not differ significantly among the three groups (*p* = 0.436 and 0.621, resp.). However, the mRNA level of PEDF was significantly higher in the PDR and CRVO groups than in the control group (*p* = 0.005 for PDR versus POAG groups and *p* = 0.045 for CRVO versus POAG groups). The difference in the mRNA level of PEDF was not significant between the PDR and CRVO groups (*p* = 0.584, Figure 1).

**Figure 2:**
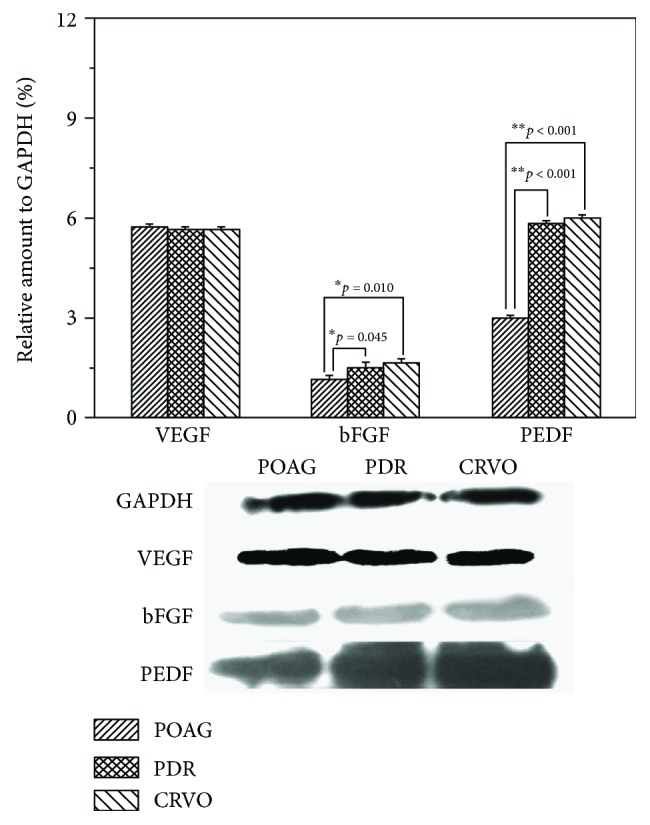
Protein levels of vascular endothelial growth factor (VEGF), basic fibroblast growth factor (bFGF), and pigment epithelium-derived factor (PEDF) measured through Western blotting and normalized to the glyceraldehyde-3-phosphate dehydrogenase (GAPDH) level. VEGF levels did not differ among patients with neovascular glaucoma (NVG) secondary to proliferative diabetic retinopathy (PDR), central retinal vein occlusion (CRVO), and primary open-angle glaucoma (POAG). bFGF and PEDF levels were significantly higher in patients with NVG secondary to PDR and CRVO than in patients with POAG (control) (*p* = 0.045 and 0.010 for bFGF, resp.; all *p* < 0.001 for PEDF). No significant difference was found between the two NVG groups (*p* = 0.738 and 0.322 for bFGF and PEDF, resp.).

The results of the Western blot analysis indicated that the protein level of VEGF did not differ significantly among the three groups (all *p* > 0.05); however, the levels of bFGF and PEDF were significantly higher in both the PDR and CRVO groups than in the control group (*p* = 0.045 and 0.010 for bFGF, respectively; both *p* < 0.001 for PEDF). The protein levels of bFGF and PEDF did not significantly differ between the PDR and CRVO groups (*p* = 0.738 for bFGF; *p* = 0.322 for PEDF, Figure 2).

## 4. Discussion

Neovascularization is a complex process in which many pro- and antiangiogenic factors are involved [7, 11]. Among these factors, VEGF, bFGF, and PEDF are the three most crucial cytokines that participate in retinal neovascular diseases [3, 4]. Studies have reported an increase in the VEGF level in the aqueous humor of patients with age-related macular degeneration, diabetic retinopathy, CRVO, and NVG [12, 13]. In patients with NVG, the intraocular VEGF level has been reported to be associated with not only intraocular pressure control but also surgical success rates and visual outcomes [13–16]. Therefore, intravitreal anti-VEGF therapy has now become one of the mainstream treatments for NVG. In patients with NVG, tangential traction and mechanical obstruction of the trabecular meshwork by the fibrovascular tissue on the iris play a crucial role in the pathogenesis of NVG [17, 18]. bFGF is one of the other crucial regulators suggested to be involved in tissue fibrosis. In addition, bFGF has been shown to enhance endothelial cell proliferation, migration, and angiogenic differentiation [4, 19].

PEDF can be identified and isolated from retinal pigment epithelial cells and has been found to have potent antiangiogenic activity. PEDF expression has been indicated to be negatively correlated with VEGF expression in the aqueous humor and blood of patients with PDR [20–24]. Elayappan et al. reported that PEDF can inhibit the migration and tube formation of retinal endothelial cells in the presence of VEGF [25]. However, Matsuyama demonstrated no change in the intraocular PEDF level after the administration of anti-VEGF agents intravitreally [26].

Theoretically, a tissue would express and secrete a protein through the translation and synthesis of mRNA. If the protein and mRNA levels of the tissue have both been elevated, then that protein would be considered to be secreted by the tissue itself. However, if the protein level has increased but the mRNA level has remained low, as in a control, then the tissue might have been influenced by this protein, and the tissue itself might not have secreted the protein. In the present study, we analyzed the mRNA and protein levels of the three cytokines and found that the mRNA and protein levels of VEGF were not significantly higher in the irises of the patients with NVG associated with PDR or CRVO than in those of the patients with POAG. This finding can be attributed to the preoperative injection of ranibizumab, which may have markedly downregulated the intraocular protein level of VEGF. However, the equivalence of mRNA levels suggests that the neovascular iris tissue of the patients with PDR and CRVO did not itself express excessive VEGF.

The protein level of bFGF was significantly higher in the patients with NVG than in the patients with POAG. This finding signifies that the increase in the bFGF level may play a role in the neovascularization of the iris in patients with PDR or CRVO. However, the mRNA level of bFGF in the patients with PDR and CRVO was similar to that in the patients with POAG. Thus, it can be hypothesized that the increased bFGF protein was expressed by and diffused from the posterior segment of the eyeball, mainly the retina, and was not secreted by the iris stroma. This finding demonstrates that iris neovascularization in patients with NVG secondary to retinal diseases is primarily caused by proangiogenic cytokines triggered or expressed by only the ischemic retina.

In this study, we found that the mRNA and protein levels of PEDF were significantly higher in the irises of the patients with NVG than in those of the patients with POAG. This result suggests that not only the retina but also the iris itself may be able to produce this antiangiogenic factor. Although the protein level of VEGF was not high in our study due to anti-VEGF therapy, the overexpression of the mRNA and protein levels of PEDF in the iris tissue might have constituted a negatively controlled process that balanced the persistently increasing levels of angiogenic factors in the anterior chamber. The iris tissue did not overexpress proangiogenic factors, even in the patients with PDR. Because the iris has complex blood circulation and higher blood flow than that in the retina, the iris tissue may more easily receive sufficient oxygen or nutrition and seldom triggers the expression of these cytokines.

This study has several limitations. First, we compared the mRNA and protein levels of the patients with NVG with only those of the patients with POAG, rather than with those of the normal population. Although we excluded patients with any other possible mechanisms for glaucoma from the control group, no evidence supports that VEGF, bFGF, and PEDF expression in the irises of patients with POAG is similar to that in the irises of healthy people. Additional studies comparing representative controls should be conducted to confirm our results. Second, our study examined only three typical angiogenic factors, which may not represent all changes that occur in the irises of patients with NVG. More angiogenic and inflammatory cytokines should be analyzed to verify our conclusions. Third, in this study, we hypothesized that the protein levels in the iris tissue are highly correlated with those in the aqueous humor. However, future studies should examine the protein levels of various cytokines in aqueous or vitreous specimens and compare them with the levels in the iris tissue. Fourth, the small sample size of this study led to a relatively large standard deviation in our results. For example, the mRNA level of PEDF of the two patients in the CRVO group was considerably lower than the other data in the same group, which resulted in a low mean value with high standard deviation in the results. Although we found statistical significance, the clinical significance of this variation should be considered, and an additional study with a larger sample size should be conducted. Finally, after anti-VEGF treatment, the situation inside the eye may become more complex than that after the treatment of naïve eyes.

In summary, our study results reveal that the protein levels of bFGF and PEDF were elevated in the irises of the patients with retinal-disease-associated NVG. The protein levels of VEGF in these patients were similar to those in the patients with POAG 7 days after intravitreal injections of ranibizumab. Increased mRNA levels of PEDF were found in the irises of the patients with NVG. By contrast, the mRNA levels of bFGF were not increased in the irises of the patients with NVG. Our results suggest that the iris tissue itself may secrete PEDF but not bFGF in these diseases. The treatment of these patients should focus on the origin of proangiogenic cytokine expression rather than the neovascular iris.

## Figures and Tables

**Table 1:** Primers for quantitation of VEGF, bFGF, and PEDF.

Target	Primer's sequence (5′-3′) (forward and reverse) and length (bp)	Amplicon size (bp)
VEGF	(F) AGATCGAGTACATCTTCAAGCCATC (25)	66
	(R) CGTCATTGCAGCAGCCC (17)	
bFGF	(F) CCGACGGCCGAGTTGAC (17)	112
	(R) TAACGGTTAGCACACACTCCTTTG (24)	
PEDF	(F) CGACCAACGTGCTCCTGTCT (20)	131
	(R) GATGTCTGGGCTGCTGATCA (20)	
GAPDH	(F) CATCCATGACAACTTTGGTATCGT (24)	74
	(R) CAGTCTTCTGGGTGGCAGTGA (21)	

VEGF: vascular endothelial growth factor; bFGF: basic fibroblast growth factor; PEDF: pigment epithelium-derived factor; GAPDH: glyceraldehyde-3-phosphate dehydrogenase.

**Table 2:** Summary of clinical findings (mean ± standard deviation).

Group	Total	POAG	NVG secondary to PDR	NVG secondary to CRVO	*p* value
Age	50.88 ± 10.76	54.38 ± 8.50	50.13 ± 8.97	48.13 ± 12.80	0.378^∗^
Gender (male/female)	23/16	6/6	8/4	9/6	0.641^∗∗^
BCVA (logMAR)	2.15 ± 1.18	1.27 ± 0.92	2.59 ± 1.33	2.59 ± 0.79	0.018^∗^
IOP at the time of surgery (mmHg)	37.04 ± 13.25	33.38 ± 14.54	38.63 ± 14.67	39.13 ± 11.24	0.358^∗^

We included 39 eyes from 39 patients (12 eyes from 12 patients with POAG, 12 eyes from 12 patients with NVG secondary to PDR, and 15 eyes from 15 patients with NVG secondary to CRVO). ^∗^One-way ANOVA; ^∗∗^*χ*^2^ test. NVG: neovascular glaucoma; POAG: primary open-angle glaucoma; PDR: proliferative diabetic retinopathy; CRVO: central retinal vein occlusion; BCVA: best-corrected visual acuity; IOP: intraocular pressure.

## Data Availability

The data of this study can be obtained in the medical database of People's Hospital of Peking University.

## Additional Points

*Summary Statement*. Retinal-disease-related neovascular glaucoma is a vision-threatening condition. The authors measured the messenger RNA (mRNA) and protein levels of cytokines and found that the basic fibroblast growth factor and pigment epithelium-derived factor (PEDF) were mainly expressed in other tissues rather than the iris. On the other hand, the iris might express PEDF because its mRNA level in the iris was high.
